# “With fever it’s the real flu I would say”: laypersons’ perception of common cold and influenza and their differences - a qualitative study in Austria, Belgium and Croatia

**DOI:** 10.1186/s12879-018-3568-9

**Published:** 2018-12-12

**Authors:** Elisabeth Anne-Sophie Mayrhuber, Wim Peersman, Nina van de Kraats, Goranka Petricek, Asja Ćosić Diviak, Silvia Wojczewski, Kathryn Hoffmann

**Affiliations:** 1 Department of General Practice and Family Medicine, Center for Public Health, Medical University of Vienna, Kinderspitalgasse 15, 1090 Vienna, Austria; 2 Department of Social Care, Odisee University College, Brussels, Belgium; 3 Department of Physical Therapy and Motor Rehabilitation, Ghent University, Ghent, Belgium; 4 Department of Family Medicine, School of Medicine, University of Zagreb, Zagreb, Croatia; 5 “Zagreb-Centar”, Health Center, Zagreb, Croatia; 6 Institute of Geography and Sustainability, University of Lausanne, Lausanne, Switzerland

**Keywords:** Influenza, Common cold, Laypersons, Differences, Austria, Belgium, Croatia

## Abstract

**Background:**

There is little research on laypersons’ perceptions regarding common cold and influenza, their symptomatic distinction and considerations of risk. This study investigates understanding of pathogenesis across three European countries and provides a knowledge base from which adequate prevention recommendations and treatment advice can be derived.

**Methods:**

This is a qualitative research study. Semi-structured face-to-face interviews were conducted with 85 participants from three European countries (Austria *n* = 31, Belgium *n* = 30, Croatia *n* = 24) about their experiences, perceptions and risk considerations regarding the common cold and influenza. We performed a qualitative thematic content analysis.

**Results:**

Three main themes were identified: common cold as harmless with individualistic symptoms; influenza as mainly distinguishable by fever, confinement to bed and severity of symptoms, but description about onset and duration are diverse; and views on pathogenesis contain references to disease causing agents and circumstances. Overall we found that risk perception is based largely on personal experience and risk is assumed moderate for both diseases.

**Conclusions:**

Study participants possessed a fairly good understanding of symptoms, differences and pathogenesis of common cold and influenza; but explanations integrated misconceptions, such as misinterpretation of fever, disease continuums, diverse onset ideas etc. Perceptions were largely based on lived experiences and interventions for prevention and treatment should be led by health care workers and focus on these issues. Basic consultations, awareness raising activities and other knowledge disseminations strategies should include aspects of communicableness and the self-limiting nature of both diseases. An informed understanding of both infectious diseases is crucial and may also increase influenza vaccination coverage in the three respective countries effectively.

## Background

The common cold is an acute infection of the upper respiratory tract, and is recognized worldwide as the most common reason for illness [1–5]. Symptoms are caused by more than 100 different viruses [6] including rhinoviruses, adenoviruses or parainfluenzaviruses [7, 8]. Common colds are self-limited and typically resolve within ten days. Secondary bacterial infections are less common: a study by Lingard et al. showed that secondary bacterial infections occurred in only about 8% of patients [9]. Symptoms can include coryza, cough, sore throat, headache, fever, and myalgia. Treatments are typically targeted towards symptomatic relief [3–5]. In contrast, influenza is caused by influenza viruses and is much more often linked to secondary bacterial infections, such as bacterial pneumonia, though, influenza is normally also observed to be a self-limiting disease with a two to three week duration. Complications of influenza can include myocarditis, or otherwise worsening of existing chronic pulmonary or cardiopulmonary diseases. The number of worldwide cases of severe illness caused by influenza each year is estimated to be in the range of three to five million, and among this there occur about 250,000 to 500,000 deaths [10]. High risk groups include those older than 65 years as well as children under five [11]. Influenza is manly recognized as starting with onset of a sudden high fever, whereas a common cold more commonly begins with coryza or a sore throat [2, 11]. Although from a microbiological point of view the two diseases are clearly different from each other as distinct viruses are involved [2, 6, 12], it may be difficult to differentiate the diseases for lay persons because they share several symptoms such as fever, cough, or limb ache. This overlap has been observed to increase the risk of confusion not only in lay persons but also in clinicians [2, 6, 13]. In contrast to previous publications which are dealing either with common cold or influenza [1, 6, 12, 25], there have been few publications about the clinical differentiation between common cold and influenza [2, 13] with very little research done on peoples’ perceptions about them. Public health and primary care studies have repeatedly shown that strategies for the improvement of the health of a population essentially rely on the inclusion of experiences and perceptions of target populations [13, 14].

In addition and also contrary to the majority of previous publications, we included three countries in our study. These three countries are located in different geographical regions of the EU to be able to explore cross-country variations as fairly as possible. The aim of this study is to explore how individuals across Europe, namely in Austria, Belgium and Croatia perceive and recognize the difference between a common cold and influenza. The objective is to gain an understanding of laypersons’ reasoning, concept of knowledge and descriptions as well as investigate cross-country variations.

## Methods

### Design

This study is designed as a qualitative research study and includes participants from urban and suburban parts from the Eastern part of Austria [15], from Flanders, the Dutch speaking part of Belgium and from Zagreb in Croatia. Data collection was conducted between November 2013 and June 2014 in Austria, February 2016 and April 2016 in Belgium and March and July 2016 in Croatia using semi-structured interviews as well as a short quantitative questionnaire for socio demographic data (designed for this study). The interview guide and the short questionnaire were translated for each setting. Qualitative methods help to illustrate social realities of the people, as the researcher engages face-to-face with the respondent and allows elucidating respondents’ reasoning and explanations. During the interview process the interviewer had the chance to adapt questions from the interview guide in order to make it more comprehensive to the respondent [16, 17].

### Participants

In all three countries purposive sampling was applied to recruit participants, following pre-determined criteria, with slightly different approaches in the respective country. In Austria and Belgium, participants were recruited from the general population by the interviewers themselves according to inclusion and exclusion criteria. In Austria about half came from the urban part of Vienna while the other participants had a rural background and were recruited in Lower Austria [18] (a total of 31 participants). In Belgium, all participants were living in Flanders (a total of 30 participants). The study in Croatia was conducted in a general practice setting, six general practitioners (GPs) were conveniently selected from the health centre “Zagreb-Centar” (three from urban and three from the suburban area of Zagreb) to recruit the patients. Each GP recruited four patients from their list (a total of 24 participants).

Participants were contacted and informed about the purpose of the study and study design prior to the interview (in Austria and Belgium by the interviewer, in Croatia by his/her GP). The participants were invited to participate and required to sign a written consent form before participation. Only one patient (from Croatia) refused because of lack of time and was replaced by another patient from the respective doctor’s patient list. The small quantitative questionnaire on socio demographic data was filled in by all participants and collected information on gender, age and level of education.

#### Inclusion criteria

Participants had to be at least 18 years old, physically and psychologically able to participate in the study, had to be able to communicate in the respective local language with the interviewer and live in Vienna or Lower Austria (Austria), Flanders (Belgium) and urban or suburban area of Zagreb (Croatia).

#### Exclusion criteria

Participants were excluded if they worked in a health related field, in Belgium also if their family studied for, or worked as, health workers.

### Data collection

In Austria, two medical diploma students (CS and FK) trained in qualitative methods, conducted the interviews and were supervised by KH. In Belgium, two master students in Health Education and Health Promotion (NvdK and AV) trained in qualitative methods conducted the interviews and were supervised by WP. In Croatia, family medicine vocational trainee trained in qualitative methods (ACD) carried out all interviews and was supervised by GP. All interviews lasted from 15 to 45 min at a place of the participant’s choice and were held in respective local language (German, Dutch or Croatian). All 85 transcripts met Kvale’s quality assurance criteria and were used for the analysis [19].

### Data analysis

All interviews were recorded and transcribed verbatim and transcripts were checked for accuracy by the authors of this paper, from the respective country. The transcripts were imported to the Atlas.ti or NVivo software for qualitative data analysis [18, 20–22]. One researcher per country (EAM, NvdK and ACD) performed a qualitative content analysis coding inductively and deductively according to the research questions.

Thereby we followed Pope et al. to explore the data inductively using content analysis to generate categories and explanations [23]. The code generation was in accordance to the questions in the interview guide “*How do you differentiate the common cold from influenza?*” Another relevant aspect analysed was respondents’ considerations on risk, and what they identified as reasons or causal factors for both illnesses. The codes were summarized and discussed with the other authors of this paper, first within the country (for Austria: EM, KH, SW; for Belgium: WP, NvdK; for Croatia: ACD, GP, and: ZOA, VC), subsequently with the authors of the other countries. The results of Austria were used as the starting point for the discussion of the transnational results. Original quotes were translated into English and are presented in the results section.

### Ethical statement

The authors guarantee that the study was conducted according to the guidelines of the “Declaration of Helsinki”. Moreover, the study received a positive vote from the Ethics committee from the Medical University of Vienna (EK 1786/2013), Ghent University Hospital (EC/2016/0185 and EC/2016/0283), Research Ethics Committee of the “Zagreb-Centar”, Health Center (251–510–03-20-16-08).

## Results

### Sample characteristics

In total *n* = 85 persons between the ages of 18 and 83 participated in this study (see Table 1). In the questionnaire the level of education was classified according to country-specific differences but for reasons of comparability mapped out in the present study in two categories only. In Belgium interviews were conducted with a significantly larger amount of people with higher education background, this difference was, however, not reflected in results.

**Table 1:** Sample characteristics

Age	Austria	Belgium	Croatia
18–30	6	14	9
31–45	6	4	2
46–60	7	9	4
61–75	6	2	7
76+	6	1	2
Gender			
female	19	16	10
male	12	14	14
Level of education			
Higher education	2	16	3
No higher education	29	14	24
Influenza vaccination in the year of interviewing			
yes	5	6	5
no	26	24	19

### Terminology and wording

In this paper the term *common cold* is used to refer to *colds*, *chills* and *flu-like infections*, while *influenza* specifies the diseases caused by the influenza virus, commonly known as *the flu* or *the real flu*.

### Common cold and influenza

#### Use of terms

Interview descriptions contain different references to illnesses. We found that across the three countries some respondents rather describe general suffering and provide descriptions about specific symptoms, than identify them to one singular associated illness. Other respondents distinctly identify one illness and describe *the common cold* or *the flu* and its distinctive symptomatic, risk and features. The following analysis integrates all relevant views and perceptions, around the three main themes: [1] perception of common cold as harmless and individualistic disease, [2] influenza and its distinctive characteristics and [3] perception of pathogenesis and disease progression.

#### Perception of common cold as harmless and individualistic disease

Most respondents perceive a common cold as benign, rather harmless or mild disease, and describe it as “nothing dreadful”. Typically described symptoms include getting the sniffles or a stuffy nose, nasal congestion, coughing and sneezing, and a sore throat. Also, but less frequently, headache, bodily aches, fatigue and a feeling of weakness were mentioned. Some respondents associate symptoms and their severity with their individualistic, physical health circumstances, their susceptibility and, therewith related, their immune system. Personal health problems play a significant role in disease description, some describe themselves as particular types, others explain that they themselves exhibit for instance a particular proneness to headaches, to sinuses infections etc.

*“With me it’s nose, throat and coughing (…) When I have a cold, well so for me it depends on how much my nose is affected, this is now really specific (…) with me it is the nose because I don’t get the typical sniffles but before that I get this burning in my sinuses” (A16, female)*

It was found that the majority of respondents in Austria explain no fever, a slight fever and a feverish feeling to be all equally typically, or potentially, occurring in the event of a common cold, whereas in Belgium and Croatia the majority of participants do not link fever to common cold. Overall, for most participants we found that high fever was in fact frequently and definitely viewed to be pathognomonic to influenza and not to common cold.

In the opinion of respondents, the severity of the symptoms and the individualistic health condition significantly determines if there is a need for treatment and for adapting the daily routine. If it was experienced as benignant respondents explain to typically follow their daily routine, if it is more severe treatment is necessary. Occasionally, respondents emphasise more need for resting or the inability to concentrate.

*“Common cold is when you feel weak; you have a runny nose; sore throat and cough (…) you can still function reasonably, if you can, you get some rest for a day or two, but still you can go to work and everything. So, you don’t feel good, but it is not, how would you say, a disaster.” (C9, male)*

Respondents also have a fairly individualistic conception of the onset of a common cold. Thus, some respondents describe the sequence of symptoms to occur in a particular order: “the coughing comes after the stuffy nose, every person is different”, “with me it always starts with throat and coughing”, “I notice it (ref. cold), with me always like, it is always the tonsils that are swollen a bit more shortly beforehand” or, “it is always the initial sign that it hurts back there (…) a headache and a stuffy nose, I always get a fever blister”. Others associate the feeling of weakness, dizziness, exhaustion, tiredness, light sensitivity, difficulty to concentrate, increased need to sleep and hoarseness with the onset of a cold. The symptom onset of a common cold described by most participants as gradual, but some also say it occurs abrupt. Some symptoms of common cold are mentioned only once or twice, such as a lack of appetite, little energy, ear pain, sputum, heavy sweating, diarrhoea and overall problems with mucous membranes. Most participants relate the common cold disease to their individual experiences and explain it lasts between a few days to around a week.

### Influenza and its distinctive characteristics

Influenza is understood as severe, long-lasting and less frequently occurring in comparison to common cold. The symptoms most mentioned are fever and heavy sweating, fatigue, weakness and aches, particular limb aches. The main distinction between common cold and influenza in the interviews revolves around three dimensions. The first one is fever, most of the respondents’ state that they classify *the flu* as a “fever disease” and assume that in the event of fever it is *the actual flu*. In contrast to the absence of fever which was identified as a marker for merely a common cold infection, see above. Some respondents do not mention fever as a sign at all or are unclear about what medical condition that can cause fever. Therewith related, an additional distinction of influenza is the stronger need for resting and in some cases full confinement to bed, outlined as “you do not feel able to go to work, school or university” (A11), “you can’t do sports” (A27) or experiences as more dreadful “if you are half unconscious lying there” (A20), “you cannot move” (A28). The third distinction is the experienced severity of symptoms; influenza is primarily associated with more, more intense aches and entire body pains.

Some respondents explain that they are unsure about the actual differences between the diseases and some make vague assumptions about seasonal occurrences of one or the other.

*“Well I couldn’t really tell [note: the difference] as I never had the real influenza. I only had limb aches, if that is a sign for influenza?” (A14, male)*

Influenza onset is more frequently classified as abrupt. The conception of influenza symptoms entail more often what could be called “whole-body-symptoms”, associated with limb pains or entire feeling of weakness:

*“How does it start, well (…) in a way you feel it in your whole body, sniffles you only feel in your head, the head hurts, but the flu you fell all your limbs” (A22, female)*

### Perception of pathogenesis and disease progression

Ideas about pathogenesis provide instructive insights into participants’ reasoning. In cases where influenza was primarily considered as a severe form of a common cold that “knocks you out”, links to other known diseases like angina, sinusitis and bronchitis were frequently made, but a differentiation between the diseases was absent. Only a few respondents refer to the influenza virus as a distinctive factor for influenza, “a flu virus is simply more severe than a flu-like disease”. Most respondents do not point to viral pathogens and do not referred to the existence of different viral pathogens. However, some identify a distinction between viruses and bacteria but state that they are unsure about what constitutes the difference; others state that there may not be a difference at all. Only a few respondents stated specifically that influenza cannot be treated with antibiotics, considering it is a viral infection.

The majority of respondents referred to the possibility of infection or the communicableness of both diseases. While respondents very rarely mention “the wave of influenza” they explain common colds as well as influenza to be seasonal-dependent. Thus, external circumstances including interchange of seasons, lower environmental temperatures, winds/drafts, the transition periods, damp weather conditions, are identified as most typical times to fall ill. Respondents also mention inappropriate wardrobe and cold foods and drinks to enable catching a cold.

With the exception of Belgium, influenza is reported to occur not every year; while common cold is typically estimated to be experienced once, twice or three times a year. Almost all respondents declare that they have some experience with influenza, because they experienced influenza first hand, or for instance through family members or friends. Several participants from Austria assume that they “probably” have never had influenza themselves. In most cases influenza experience was dated several years back, or dated last winter. In Belgium, several participants state that they have influenza once a year; while at the same time others do not remember when exactly they had it the last time.

*“I have not had that for a long time, so I really do not know anymore” (B22, male)*

In terms of disease duration, respondents estimate diverse time periods related to influenza. Some respondents state that a real flu knocks a person out for two, three weeks and that it lasts quite long, but in other statements we find periods such as “I stay three days in bed, drink tea, lemon tea, heavy sweating and the flu is gone in three days”.

With respect to the progression of disease it was noticeable that about half of the participants identify common cold and influenza as separate diseases**.** The opinion prevails that they are separate diseases, which maybe share some of the symptoms but without giving precise reasons for this assumption. However, some respondents across countries describe a possible continuum from a common cold to influenza and believe that a transition is possible.

*“I think there is a transition, because one illness is sort of a subset of the other illness and there are even further symptoms; that is why I would consider that there is a transition.” (A15, male)*

*“It often starts with a cold, I think. And yes, if you start to get a fever and your muscles begin to hurt, then it passes to the flu, it shifts a bit.” (B27, female)*

The majority of interviewed laypersons explain that disease progression of and recovery from common cold as well as influenza is linked to resting behaviour coupled with self-treatment, medication and “listening to one’s own body”. In the case of severe suffering a doctor’s visit is often recommended. Among the interviewed laypersons’ the view prevails that with a cold you are probably still able to go to work if you have to, but people still recommend to rest, stay in bed, sleep more and spare themselves. However, some participants explain that they personally do not change their routine when suffering from a common cold, “they just take it” (A2) and still go to work or school and run their errands. With a real flu, respondents explain that they would be “really affected” (health-wise) and tend to recommend to visit a doctor when it is not endurable or last longer than expected.

*“Eh, the flu. It can be very dangerous. If you miss it and you don’t go to see a doctor, it can have severe consequences.” (C10, male)*

With regard to risk perception several participants express mild to moderate concerns for themselves, but describe immunocompromised individuals, elderly and children to be potentially more vulnerable.

*“It is a sensitive issue with elderly people (…) the immune system is already weakened and a small flu-like disease can eventually turn into a lethal flu” (A12, male)*

The quotation illustrates the idea of a continuum from a flu-like disease to *a lethal flu* and essentially the identification of risk groups.

Some participants describe “well a real flu is pretty severe and potentially fatal” and respondents in Croatia refer to complications that may occur with influenza, for instance pneumonia. However, most respondents abstain from detailing the potential of a fatal influenza outcome and describe it generally “more severe”.

## Discussion

This study illustrates laypersons’ perceptions of two infections, the common cold and the influenza infection across the three European countries Austria, Belgium and Croatia. Respondents describe symptoms, distinguishable characteristics and perceptions about pathogenesis primarily based on their personal assumptions and support it by their individual experiences. Only a few participants made generalisations about diseases, medical signs and evidence-based practices and most descriptions included primarily important personal accounts of illness experiences. Surprisingly, the differences in the perception of lay persons is more divers within one country than between the countries, the overall perception in all three countries regarding [1] perception of common cold as harmless and individualistic disease, [2] influenza and its distinctive characteristics and [3] perception of pathogenesis and disease progression is similar: It is noticeable that across all three countries, we found that respondents possess a fairly accurate perception of symptoms for both diseases. At the same time results show that many are not familiar with biomedical concepts of the two diseases and it is difficult for many people to make a clear distinction. However, also clinical diagnosis of influenza is difficult because symptoms range in severity and overlap with those caused by other respiratory viruses [13].

The common cold infection is identified as generally harmless and signs and symptoms are interpreted as individualistic, depending on the person, health condition, and external circumstances. It is perceived as self-treatable, only with particularly severe symptoms participants explain to take rests, adapt their behaviour and daily routine. There were some unique positions from participants, such as that the onset of a cold is gradual or abrupt, some typical common cold symptoms were only mentioned once or twice, and that a common cold may last longer than a couple of days.

With influenza we found that respondents report more severe and longer lasting symptoms. *The real flu*, as it was mostly cited, was distinguished by fever, confinement to bed and severity of symptoms. Laypeople’s’ perception on onset and duration were vague and with regard to treatment the view prevailed that medication may very well be required if fever was involved and health seeking at a health care facility or at a GP is encouraged and necessary.

Based on the strong link on individualistic understandings of symptoms and both diseases this study found a less clear distinction between the diseases as compared to findings in Cedraisch et al. [24].

Although lay persons seem to know that influenza is more severe than common cold, the criteria which they identify with influenza concentrated more on symptom pattern: fever, limb ache, and not being able to work. This finding is similar to other publications, where laypersons were asked how they identify the flu [24, 25]. Meanwhile, fever is not always a sign of influenza [26]. In contrast, common colds can present with fever and limb ache as well. What seems noteworthy is that some respondent mentioned differences at the onset of the two diseases, although results were rather vague. The differences at the beginning of the disease (in most cases gradual onset for common cold versus sudden high fever for influenza) appear to be the best clinical criteria for the differentiation [2, 13].

Interestingly the results do no not include explanations on the self-limiting characteristic of both diseases, and that therefore the standard treatment is targeted towards symptomatic relief [1, 3, 5].

It has to be mentioned that for laypersons across the three countries the linguistic differentiation is of particular difficulty because in everyday language common cold is often referred to as “flu”, and expressions like flu-like infections (in German: “grippaler Infekt”) may suggest that it is linked. This is a common misconception in many English speaking countries as well, including the United States.

In addition, most respondents did not refer to different viral pathogens (i.e. rhinoviruses, adenoviruses, parainfulenzaviruses etc.) and infectious agents that constitute each disease. In some cases it was even stated that a transition from common cold to influenza is possible. Explanations included ideas of transitions, developments and shifts from one to the other; while relatively little emphasis is given to communicableness of both viral infections.

These perceptions are especially relevant if we take into account the issue of prevention and for instance the attitudes towards influenza vaccinations. From all participants only less than 6% (15/85) got influenza vaccination in the respective year, which is linked to perception of risk groups and attitudes towards illness, illness transition and influenza vaccination. Therefore, the hesitancy or otherwise the proactive seeking of influenza vaccination and also other preventive measures as well as treatment, seems to be a consequence of a lack of knowledge. Awareness raising activities, such as a brief explanation by a GP or practice assistance could be an effective way to counter complications with influenza, low vaccination rates and inefficient treatment pathways. Interventions for improving health literacy on the common cold and influenza disease could start with explaining the differences at their early stage and increased distribution of rapid influenza tests to ascertain to assist with management decisions. Furthermore clinicians are advised to use timely epidemiological data to ascertain if influenza is circulating in their communities [13]. Therewith related the correct wording is equally important in order to decrease the risk of confusion of the common cold from influenza. Health campaigns could easily target term distinction. Improved knowledge of lay persons about influenza could have a positive impact on influenza vaccination and information about interaction of viruses and bacteria could also be crucial to counter antimicrobial resistance. This study suggests the importance of investment in health literacy to give people the chance to gain knowledge beyond that of their own experiences with illness.

The qualitative approach of our study underpins efforts to include the voices of patients in health research as well as to improve health promotion strategies derived from actual experiences, needs and perceptions of laypersons [14]. Preventive efforts and treatment advice must respond to the particular understanding of laypersons. A crucial element of such efforts could be to explain that standard infection containment initiatives focus on encouraging individuals with influenza-like illness to stay at home, seeking medical attention only in the presence of complications [25]. This entails knowledge about the element of it being a self-limiting, self-contained disease and treatment is advisable to relief symptoms. With regards to at risk-groups, this study’s findings are crucial while confirming other studies in this regard. Laypeople identify at-risk or vulnerable groups; however, do less frequently identify themselves to be vulnerable to influenza.

The study has some limitations. First, the participation of our respondents was voluntary, leading to the possibilities for selection bias. This means that respondents who have more time, are more interested in the topic, or think that they want to deal with this issues also through giving an interview are represented to a higher degree. Thus, understandings and perceptions that are described in this study may not reflect what average individuals understand as common cold, influenza and its differences. It is possible that individuals who in fact may be more affected by decisions about common cold and influenza treatment (e.g. children, elderly people) may have a particular perception on these issues that was not covered by this manuscript. Another limitation is that some interviews were very short. Because interview guidelines were slightly adapted according to each interview, our material provides an in-depth view into certain themes and touches other themes only rudimentary. This is a limiting factor, data analysis was driven by the available data that was collected, further studies would benefit from longer, more in-depth interviews and possible follow up interviews with respondents. The data is based on self-reports by participants, thus being subject to possible recall bias.

Another limitation is the fact that the sampling period in Austria (influenza season 2013/2014) is different from the one in the other two countries (influenza season 2015/2016). Fortunately, both influenza seasons were mild influenza activity seasons in Europe [27, 28] leading to the assumption that this factor does not have a bigger impact on the comparative results between the countries. However, since both seasons were mild the perception of lay persons regarding influenza might have been less alerted and kept in mind when it came to the different lay-concepts of the two diseases common cold and influenza.

## Conclusions

We conclude that participants possessed a fairly good understanding of symptoms, differences and pathogenesis of common cold and influenza. While at the same time we found some existing misconceptions regarding fever as an indicator of influenza, some vague assumptions about gradual and/or abrupt onset of common cold, and the idea of a possible continuum from one disease to the other. There were very few cross country differentiations in results. Interventions for prevention and treatment should focus on misconceptions and misinterpretations and awareness raising activities as well as other knowledge dissemination strategies should be led by health care workers. All first point of contact actors, may it be physicians, GPs and practice assistants, should be strongly supported and incentivised in their role as knowledge mediators. Basic consultations and awareness raising activities must include biomedical explanations of infectious disease pathogenesis, focussing on symptom onset differentiation, and also include aspects of communicableness and the self-limiting nature of both diseases. An informed understanding of both infectious diseases is crucial and may also increase influenza vaccination coverage in the three respective countries effectively.

## Abbreviations

Atlas.ti: The Qualitative Data Analysis and Research Software
EU: European Union
GP: General Practitioner
NVivo: A qualitative data analysis computer software package
