# Correction to: “With fever it’s the real flu I would say”: laypersons’ perception of common cold and influenza and their differences - a qualitative study in Austria, Belgium and Croatia

**DOI:** 10.1186/s12879-019-4199-5

**Published:** 2019-06-26

**Authors:** Elisabeth Anne-Sophie Mayrhuber, Wim Peersman, Nina van de Kraats, Goranka Petricek, Asja Ćosić Divjak, Silvia Wojczewski, Kathryn Hoffmann

**Affiliations:** 1 Department of General Practice and Family Medicine, Center for Public Health, Medical University of Vienna, Kinderspitalgasse 15, 1090 Vienna, Austria; 2 Department of Social Care, Odisee University College, Brussels, Belgium; 3 Department of Physical Therapy and Motor Rehabilitation, Ghent University, Ghent, Belgium; 4 Department of Family Medicine, School of Medicine, University of Zagreb, Zagreb, Croatia; 5 Zagreb-Centar, Health Center, Zagreb, Croatia; 6 Institute of Geography and Sustainability, University of Lausanne, Lausanne, Switzerland

**Correction to: BMC Infectious Diseases (2018) 18:647.**

**https://doi.org/10.1186/s12879-018-3568-9**

Following publication of the original article [1], the authors reported an author name has been misspelled. Asja Ćosić Diviak should be replaced with Asja Ćosić Divjak.
